# Epigenetic Mechanisms in Canine Cancer

**DOI:** 10.3389/fonc.2020.591843

**Published:** 2020-10-23

**Authors:** Pedro Luiz Porfirio Xavier, Susanne Müller, Heidge Fukumasu

**Affiliations:** ^1^ Laboratory of Comparative and Translational Oncology (LOCT), Department of Veterinary Medicine, Faculty of Animal Science and Food Engineering, University of Sao Paulo, Pirassununga, Brazil; ^2^ Structural Genomics Consortium and Institute of Pharmaceutical Chemistry, Buchmann Institute for Molecular Life Sciences, Johann Wolfgang Goethe University, Frankfurt am Main, Germany

**Keywords:** comparative oncology, epigenetics, DNA methylation, histone modifications, canine cancer, non-coding RNAs

## Abstract

A plethora of data has highlighted the role of epigenetics in the development of cancer. Initiation and progression of different cancer types are associated with a variety of changes of epigenetic mechanisms, including aberrant DNA methylation, histone modifications, and miRNA expression. At the same time, advances in the available epigenetic tools allow to investigate and reverse these epigenetic changes and form the basis for the development of anticancer drugs in human oncology. Although human and canine cancer shares several common features, only recently that studies emerged investigating the epigenetic landscape in canine cancer and applying epigenetic modulators to canine cancer. This review focuses on the existing studies involving epigenetic changes in different types of canine cancer and the use of small-molecule inhibitors in canine cancer cells.

## Introduction

The epigenome consists of a set of complex, dynamic, and reversible information comprising chemical modifications of the DNA and histone proteins, which are directly associated with the regulation of gene expression within the genome. These modifications, described as “Epigenetic Marks”, are heritable and occur without changes in the DNA sequence, playing a key role in biological processes such as embryonic development, differentiation, gene imprinting and silencing of the X chromosome. Furthermore, epigenetic modifications can affect DNA accessibility having a major influence on DNA-based processes including transcription, DNA repair, and replication (1).

**Figure 1:**
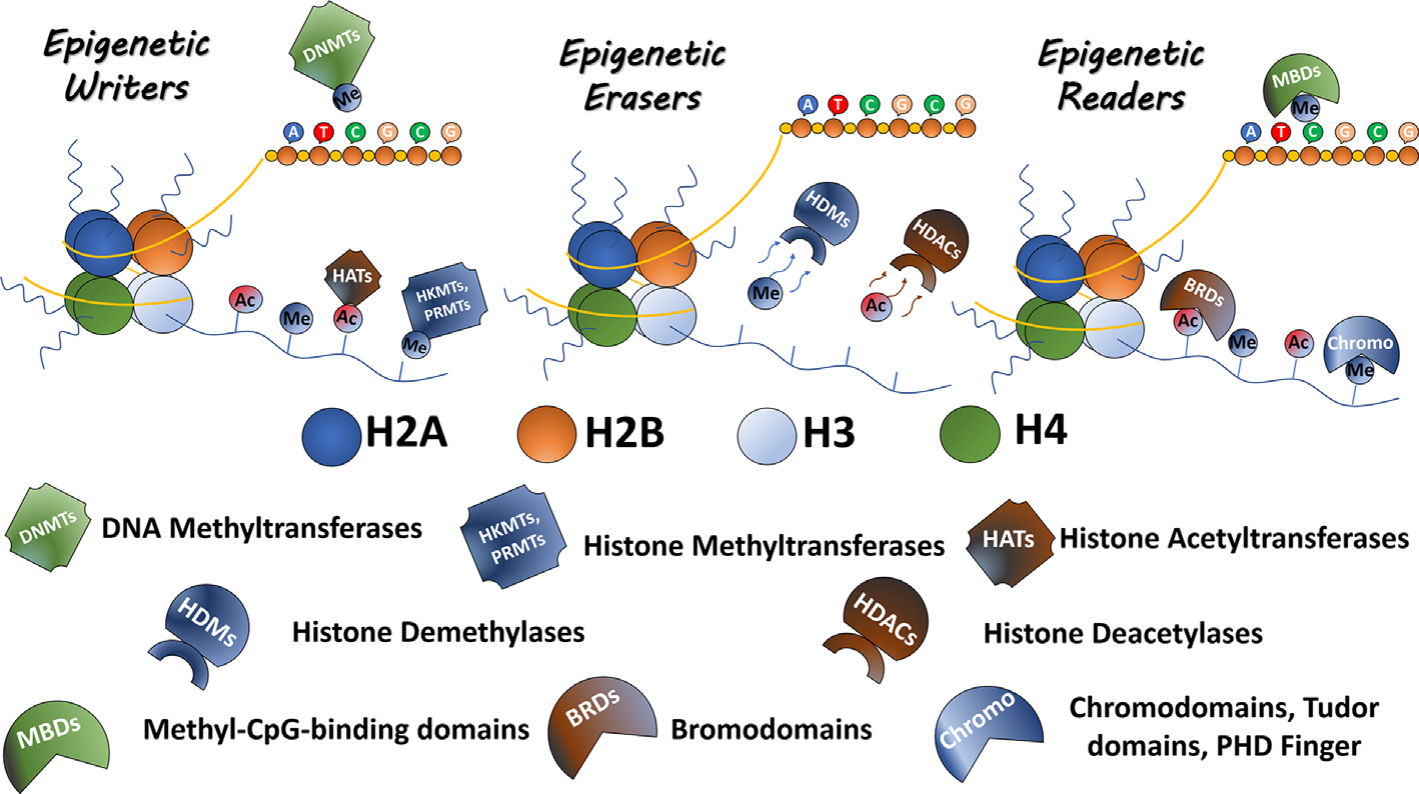
Epigenetic modulators. The epigenetic proteins are classified as Readers Writers and Erasers. Writers including DNMTs, HKMTs, PRMTs, and HATs are responsible to mark residues in DNA or histone tails. Erasers, including HKDMs and HDACs, remove epigenetic marks. Readers such as proteins containing bromodomains, chromodomains, Tudor domains, and PHD fingers recognize and bind to the epigenetic marks.

The epigenetic process is driven by a machinery of proteins, responsible for adding, removing or recognizing modifications of the DNA and histones. Epigenetic ‘writers’ are responsible for adding epigenetic marks and include DNA and histone methyltransferases as well as histone acetyltransferases (HATs); the ‘erasers’ such as histone deacetylases (HDACs) and histone demethylases (HKDMs) remove the corresponding epigenetic marks. Finally, a class of proteins exists, which recognizes and ‘interprets’ epigenetic modifications, referred to as ‘readers,’ a large class of proteins with reader domains for residues such as acetyllysine residues, including bromodomains, and methyllysine residues, such as chromodomains, Tudor domains, PhD domains, and others (2) (**Figure 1**). All these proteins play important functions in regulating gene expression, acting directly on DNA accessibility or indirectly recruiting non-coding RNAs and chromatin remodelers (3). Therefore, abnormal expression or mutations in these chromatin regulators can alter the pattern of gene expression and, consequently, lead to the induction and maintenance of diverse types of diseases, including cancer. Besides, these epigenetic alterations represent disease biomarkers with diagnosis and/or prognosis potential (4). Several of these epigenetic protein classes have been shown to contain druggable targets opening up possibilities to treat epigenetic-associated diseases (5–7).

The dog is probably the best model for human disease and has several advantages in comparison to other animal models, such as natural development of several different tumors similar to humans; generally shares the same environment and exposure to the same carcinogens as humans thereby influencing the epigenetic make-up (8). However, in comparison to human cancer, the number of studies investigating the epigenetic landscape in canine cancer is still restricted and the potential of epigenetic drugs in the treatment of canine cancer remains widely unexplored. In this review, we highlight epigenetic studies of canine cancer and provide information about how alterations of epigenetic regulators can influence diverse types of canine cancer. We also highlight potential drugs aimed at targeting these epigenetic regulators.

## An Overview of Canine Cancer

Over the past years, the animal-owner relationship has been changing and pets have genuinely become part of the human family. Therefore, advances in veterinary care have emerged, and, just like in humans, an increased life expectancy of dogs is observed (9). Consequently, age-related diseases, mainly cancer, are becoming the main causes of deaths in dogs worldwide (10–15). The epidemiologic studies of canine cancer are largely retrospective and usually present varying results depending on the region where they were performed. The incidence of cancer in dogs is 99.3 per 100,000 in male dogs and 272.1 in female dogs according to a study performed in Genoa, Italy (16). A large set of studies has shown that most common types of canine tumors are located in the skin followed by mammary tumors. Some studies have registered skin tumors frequencies as high as 40 or 50% (17, 18). However, depending on the proportion between females and males, mammary tumors have been observed around in about 36% of total cases (19), since mammary tumors account for more than 50% of the diagnosed tumors in females (20–23). Other common types of cancer in dogs are located in the soft tissues (14), hematopoietic, and lymphoid tissues (18), digestive organs (19), and bones (24).

Most canine cancer share a common pattern with the corresponding human disease including incidence, spontaneous development, associated risk factors, response to treatment, and expression of molecular targets. Non-Hodgkin lymphoma (NHL), for example, presents an incidence between 15.5 and 29.9 per 100,000 in humans and 15-30 per 100,000 in dogs (25), while mammary tumors incidence in female dogs is around 25% compared to 12% in women. Interestingly, some studies even showed that the average age at onset of mammary tumors is approximately the same for women and female dogs and the peak incidence to mammary cancer is comparable if their age is calculated proportionally (26, 27). Some strategies for cancer treatment can be applied to humans and dogs. CHOP therapy (vincristine, cyclophosphamide, prednisone, and doxorubicin), for instance, exhibits favorable outcomes in patients with lymphoma in both species, with median survival times of 8–13 months for dogs (28, 29).

Regarding genetic and signaling-pathway alterations, many types of canine cancer show similarities with their respective types of cancer in humans. Both human and canine osteosarcoma tumors carry mutations in tumor suppressor genes such as *p53* (24, 30, 31) and *RB1* (32) besides alterations in oncogenes expression including *MYC* and *MET* and constitutive expression of *STAT3* (24, 33). Overexpression of *MYC*, a consequence of copy number aberrations, can also be observed in both human and canine lymphomas (34, 35). Diffuse large B-cell lymphoma in both species exhibit alterations in NF-κB and B-cell receptors pathway signaling (36). In leukemia, a classical chromosomal rearrangement on the Philadelphia chromosome is present in 95% of cases, producing a constitutively active cytoplasmic tyrosine kinase fusion protein, BCR-ABL (37). Although less constant, the same translocation was observed in dogs with leukemia (38, 39). Canine mammary tumors and breast cancer in women share many clinical and molecular similarities, such as hormonal dependence, age of onset, and identical course of the disease (22). At the molecular level, the disease in both species also exhibits equivalent features. Despite not having a consistent molecular classification based on specific molecular markers (estrogen receptor, progesterone receptor, and *HER2*) like in breast cancer, canine mammary tumors can present germline mutations in *BRCA1* and *BRCA2* (40), important tumor suppressor genes inherited mutated in women breast tumors (41). Likewise, overexpression of *HER2* is observed between 20 and 29.7% of canine malignant mammary tumors (42), overlapping the increase of *HER2* expression exhibited by breast cancer in women (43).

Several of these similarities underscore dogs as an excellent model to study novel biological patterns and therapeutic targets in diverse types of cancer. Furthermore, the advances in veterinary oncology research has promoted and verified a substantial interest and discoveries of epigenetic alterations that assist in the development of many canine tumors, several of which are also seen in human oncology. In the following section, we highlight these epigenetic alterations and potential drugs aimed at targeting these epigenetic regulators.

## DNA Methylation and Canine Cancer

DNA methylation is probably the most studied epigenetic modification in animals and plants, playing a fundamental role in development, differentiation, and reproduction. DNA methylation occurs when a DNA n-methyltransferase (DNMT) adds a methyl group to cytosine residues in CpG dinucleotides. These CpG dinucleotides are occasionally enriched in some regions of the DNA called to CpG islands (CGIs), which in turn are preferentially located at gene promoters. DNA methylation results in the silencing of gene expression by essentially two different mechanisms: 1) DNA methylation can provide binding sites for methyl-binding domain proteins, which in turn can interact with histone deacetylases (HDACs), reducing chromatin accessibility and repressing gene activation; 2) methylation can prevent gene expression by blocking transcription factors to bind to the promoter regions of genes impeding transcription activation (44). Beyond gene promoters, CpG methylation is also found in repetitive sequences, gene bodies, and intergenic regions, which can influence gene expression of different approaches (45). CpG hypermethylation in gene bodies, for example, is associated with increased gene expression (46).

Under normal conditions, most CpG sites in the genome are methylated while the CGIs are usually unmethylated. In contrast, cancer cells exhibit a genome-wide hypomethylation and CGIs promoter hypermethylation (47). Genome-wide hypomethylation usually occurs in genomic regions including repetitive sequences, retrotransposons, and CpG poor promoters, resulting in chromosomal rearrangement, activation, and translocation of retrotransposons and, consequently, induce genomic instability. Besides, loss of methylation may lead to activation of proto-oncogenes, such as RAS, S-100, and MAGE (48). Genomic hypomethylation has likewise been observed in canine leukemia and lymphoma. In canine leukemia and lymphoma cases, 30 and 69% respectively were found to be genome-wide hypomethylated. Furthermore, these unusual methylation patterns are associated with the early phases of tumor transformation and progression in canine leukemia and lymphoma (49), just as has been observed in different types of human cancer (50–52). These findings were the first to report global hypomethylation in canine cancer and, consequently, to show similarities between the epigenetic landscape in canine and human cancer.

Also, other canine cancer types display global hypomethylation. A recent study has shown that genome-wide hypomethylation was frequently found in grade III canine mast cell tumor, which is the most common skin tumor in dogs, thus correlating DNA hypomethylation with the aggressiveness of this type of cancer (53). In addition, dogs bearing non-Hodgkin lymphoma (NHL) exhibit higher DNA global hypomethylation of circulating leukocytes in comparison with healthy dogs (54). DNA hypomethylation was also observed in canine lung cancer samples and in metastatic osteosarcoma from the primary lung cancer (55). Thus, albeit a still low number of reports, genome-wide hypomethylation seems to be a common feature of at least some types of canine cancer.

Hypermethylation of CGIs also contributes to the development and promotion of cancer through the silencing of tumor suppressor genes. In human cancer, many tumor suppressor genes such as *Rb*, *p16*, *RASSF1*, *CDH1*, *TIMP3*, and *BRCA1* have been shown to possess hypermethylated promoter regions (56). These genes are associated with processes including cell cycle, apoptosis, metastasis and DNA repair and, consequently, silencing might induce cancer. Until 2008, no article has shown the presence of promoter hypermethylation in dogs. The first report showing hypermethylation of a tumor suppressor gene in canine cancer was in canine NHL, describing the profile of DLC1 gene methylation in this cancer. DLC1 is a tumor suppressor gene found to be highly methylated in human NHL (57). Bryan and others performed methylation-specific PCR (MSP) and combined bisulfite restriction analysis (COBRA) to demonstrate the presence of DNA methylated in DLC1 in six of 13 canine NHL samples and two of three canine chronic lymphocytic leukemia, providing, for the first time, information regarding hypermethylation in canine cancer (58). In addition, there was an association between DLC1 hypermethylation and the malignant phenotype of NHL. However, hypermethylation of the DLC1 promoter was not associated with silencing of DLC1 expression and did not correlate with survival (59). Hypermethylation of *TNF-α* has been shown in human and canine melanoma cells by performing MSP. In addition, it has been observed that the methylation status and the level of *TNF-α* expression were inversely correlated in canine melanoma cell lines and melanoma tissues (60). Both human and canine melanoma cells have hypermethylated DNA in the CpG islands of the microRNA-203 (61), a suppressor of growth in melanoma cells, as shown by bisulfite sequencing and MSP (62). Hypermethylation and epigenetic silencing has also be observed for several other important tumor suppressor genes such as tissue factor pathway inhibitor 2 (*TFPI-2*) (63), death-associated protein kinase (*DAPK*) (64), cyclin-dependent kinase inhibitor 2A (*CDKN2A/p16*), *HOXD10*, *FGFR2*, *ITIH5*, and *RASAL3* in B-cell lymphoma (65–67). In addition, *DAPK* hypermethylation is associated with overall survival and considered a negative prognostic factor in canine high-grade B-cell lymphoma (68). In canine acute myeloid leukemia, a heterogeneous pattern of DNA methylation was observed with subsets of cases hypermethylated or hypomethylated when compared with healthy tissues (69). Genome-wide analysis of DNA methylation in canine lymphomas revealed that lymphoma cells have gained methylation at CpG sites located in CGIs that were unmethylated in normal peripheral blood mononuclear cells (PBMCs), used as controls. In contrast, CpG sites outside CGIs lose methylation in lymphoma cells compared to the healthy PBMCs (70).

Some evidence points to downregulation of *BRCA1*, an important tumor suppressor in mammary cancer, in canine mammary samples (71, 72). However, the mechanism responsible for the decrease of *BRCA1* expression is not well understood. Recently, a study showed *BRCA1* hypermethylation in canine mammary tumors. However, the rate of *BRCA1*-hypermethylated samples was very low (1/15, 6.7%), making it difficult to conclude that *BRCA1* downregulation is a consequence of *BRCA1* promoter hypermethylation (73). Attempts to studying both DNA methylation and histone modifications in canine cancer have been performed. Canine lymphoid tumor cell lines with different drug-sensibility were analyzed using bisulfite sequencing and chromatin immunoprecipitation, and it was found that DNA methylation and histone H3 acetylation are involved in *ABCB1* gene expression (74). *ABCB1* is a P-glycoprotein highly expressed in several different types of human cancer and has been recognized to be a key player in the multidrug resistance phenotype (75). Another study observed that CGIs of the *ABCB1* gene were hypomethylated in dogs with lymphoma. However, the authors did not find a correlation between the methylation status and levels of *ABCB1* mRNA expression in these samples (76). Both human and canine mammary carcinomas can present deregulation of estrogen receptor α (*ERα*), and its expression levels guide the prognosis and kind of therapy for the respective patient. In human breast cancer, the most aggressive type of cancer is triple-negative breast cancer, featured by the lack of *ERα* expression, which is mainly attributed to *ERα* promoter methylation (77, 78). However, no significant variation in methylation patterns were found between *ERα*-positive canine mammary carcinomas and *ERα*-negative canine mammary carcinomas pointing to a difference of *ERα* regulation mechanisms between human and dogs (79).

TET proteins are responsible to catalyze the successive oxidation of 5-methylcytosine (5mC) to 5-hydroxymethylcytosine (5hmC), 5-formylcytosine (5fC), and 5-carboxylcytosine (5caC), promoting DNA demethylation. *TET2* is considered an important tumor suppressor gene being commonly mutated in hematopoietic tumors but rarely in solid tumors (80). In hematopoietic canine tumors, *TET2* mutations have also been observed in canine mast cell tumors but in very low frequency (2.7%) (81), contrary the *TET2* mutations rate in human systemic mastocytosis, which are observed in 40% of the cases (82). *TET2* was also found mutated in canine T-cell lymphoma samples but in low frequency as well (83). All these information regarding DNA methylation and canine cancer are summarizing in **Supplementary Table 1**.

## Histone Modifications and Canine Cancer

In the nucleus, DNA is compacted and complexed by proteins called histones resulting in a DNA-protein complex named chromatin. Histone proteins can be divided in two groups: core histones (H2A, H2B, H3, and H4) and linker histones (H1 and H5). Together, these proteins make up the nucleosome (the unit of chromatin), which is ‘coated’ with 146 base pairs of DNA. Histones contain a C-terminal domain and an unstructured N-terminal domain, commonly referred to as histone tail (84). These histone tails are susceptible to different post-translational covalent modifications such as methylation, acetylation, phosphorylation, ubiquitylation, sumoylation, deamination, propionylation, and butyrylation (85, 86), which directly affect the chromatin structure by recruiting enzymes able to remodel chromatin. Consequently, histone modifications are tightly linked to cellular processes including replication, repair, and recombination (87). Furthermore, because of their influence on regulating the accessibility of chromatin to the transcriptional machinery, some modifications are responsible to regulate gene expression (88). For instance, histone acetylation neutralizes the positive charge of histone lysines and consequently results in loosening the DNA packing around histones, making it more accessible to transcriptional activation. On the other hand, histone methylation is usually associated with both transcriptional activation and repressions, depending on which residue is modified or the degree of methylation (mono-di or thimethylation).

Just as aberrations in DNA methylation patterns can lead to cancer development, altered histone modifications and chromatin changes can be observed in cancer cells. For example, some studies revealed a global loss of acetylated H4-lysine 16 (H4K16ac) and H4-lysine 20 trimethylation (H4K20me3) in different cancer cell lines and primary tumors, such as leukemia, breast, lung, and colon cancer (89). In addition, alterations of H3K9me and H3K27me patterns are also observed in different types of human cancer, including bladder, colorectal, glioma, breast, and lung cancer (90–94). Accordingly, the expression of enzymes responsible for these modifications, histone acetylases/deacetylases (HATs and HDACs) and histone methyltransferases (HMTs), is observed to be dysregulated in cancer. HDACs are often overexpressed in human cancer (95), but HATs can be also altered (96). HMTs such as EZH2 and G9a have be found overexpressed in different types of cancer (97–99).

Some studies have shown dysregulation of histone modifications exhibiting key roles in the development and progression of canine cancer (**Supplementary Table 2**). Recently, in canine urothelial carcinomas samples, significant deacetylation of histones compared to normal samples was observed, and these lower acetylation levels were associated with a poor prognosis of the animals (100). *SETD2* gene, a histone methyltransferase and an important tumor-suppressor, was found to be mutated in 21% of canine osteosarcoma samples and showed a variety of mutation types including frameshift, nonsense, splice, and missense mutations (101). Another study detects *SETD2* somatic point mutations, deletions, and chromosomal translocations in 42% of canine osteosarcoma samples (102). Just like in human cancers, overexpression of *EZH2* was found in canine lymphoma, melanoma, basal cell tumors, squamous cells carcinoma, and prostate and mammary cancer (103, 104).

Along with the simple carcinomas, complex carcinomas present the most common type of canine mammary cancer and are characterized by the presence of epithelial and myoepithelial cells (105). These types of mammary cancers seem to be profoundly influenced by the epigenetic landscape, and recent findings suggest that they originate from epigenomic rather than genomic alterations. Analysis of whole-genome sequencing, whole-exome sequencing, RNA-seq, and/or high-density arrays on twelve canine mammary cancer cases, including seven simple carcinomas and four complex carcinomas showed that, contrary to simple carcinomas, complex carcinomas did not have any copy number abnormalities and also low mutation rates. Conversely, complex canine mammary carcinomas displayed a number of epigenetic dysregulations, such as downregulation of 35 chromatin-modification genes or abnormally enriched activating histone modification H4-acetylation while showing a reduction in the repressive histone modification H3K9me3 (106).

## Non-Coding RNAs andCanine Cancer

Non-coding RNAs (ncRNAs) are defined as RNA molecules that are not translated into a protein and, for a long time, their functions in the genome were not well understood. However, with the recent advances in cell biology, transcriptomic and bioinformatic tools, it has become possible to elucidate the role of these molecules regulating biological pathways and processes. These ncRNAs are classified as microRNAs (miRNAs), transfer RNAs (tRNAs), PIWI-interacting RNAs (piRNAs), long non-coding RNAs (lncRNAs), pseudogenes, and circular RNAs (circRNAs) (107). In this review, miRNAs and lncRNAs are addressed (**Supplementary Table 3**).

**Figure 2:**
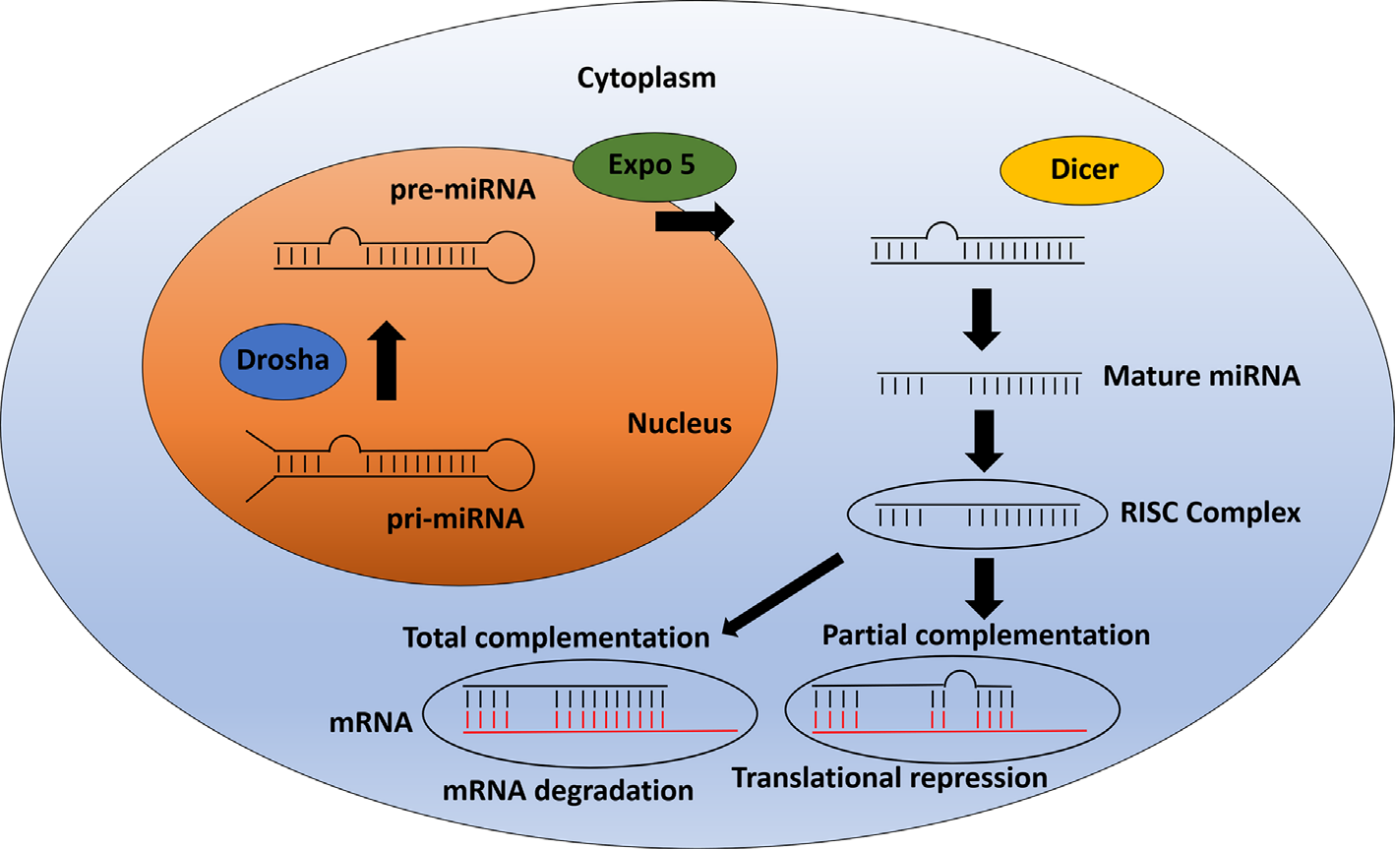
miRNA pathway: From biogenesis to mRNA inhibition. After pri-miRNA generation by transcription, the microprocessor complex Drosha processes and cleaves the pri-miRNA to produce the precursor-miRNA (pre-miRNA). Then, the pre-miRNA is transported from the nucleus to cytoplasm by Exportin 5. In the cytoplasm, pre-miRNA is processed by Dicer to produce the mature miRNA. The mature miRNA is incorporated into a protein complex termed RISC. Finally, this complex induces gene inhibition in two different ways. 1) The mRNA can be degraded if there is total complementation between the miRNA and the mRNA. 2) In the case of partial complementation, there is a translational repression.

MicroRNAs (miRNAs) are a class of non-coding RNAs encoded in the genome. The first miRNA description was in the nematode *C. elegans* (Lee et al, 1993). Since then more than 15,000 miRNAs have been identified (www.mirbase.org). Several miRNAs are expressed in many different species and are highly conserved amongst them. These molecules were found to develop a key role in many biological processes including cell proliferation, metabolism, development, differentiation, apoptosis and stress response (108–110). They control these processes by regulating gene expression through post-transcriptional mechanisms. MiRNAs bind to their target mRNA and downregulate it *via* one of two different mechanisms: 1) when miRNA and mRNA have a full complementary, the miRNA triggers the degradation of the target mRNA; 2) miRNAs can also bind to the mRNA 3’UTR regions with incomplete complementarity, leading to translational repression (111) (**Figure 2**).

Several lines of evidence have shown that miRNAs play an important role in the development of diseases in humans including cardiovascular diseases (112), neurodegenerative diseases (113) and several types of cancer. The mechanisms responsible for miRNA dysregulation in human cancer has been well elucidated and include amplification or deletion of miRNA genes, aberrant transcriptional control of miRNAs due to the dysregulation of some transcription factors such as *C-Myc* and *P53*, dysregulated epigenetic changes with some studies showing aberrant patterns of DNA methylation and histone acetylation in miRNA genes, and defects in miRNA biogenesis machinery (114–119).

The first evidence of miRNA dysregulation in canine cancer was described for canine mammary cancer. Both *miR-29* and *miR-29b* were found to be upregulated in canine mammary cancer samples. Furthermore, the same study showed that *miR-15a* and *miR-16* are significantly downregulated in canine ductal carcinomas while *miR-181b*, *-21*, *-29b*, and *let-7f* showed a significant upregulation in canine tubular papillary carcinomas (120). Thenceforth, some studies have described miRNA profiling in different types of canine cancer such as mast cell tumors (121), osteosarcoma (122), hemangiosarcoma (123), prostate cancer (124), canine multicentric lymphoma (125), and melanoma (126, 127). Interestingly, important and well-described miRNAs in human cancer including *miR-9*, *miR-18a*, *miR-126*, *miR-383*, *and miR-204* were found to be dysregulated in canine cancer. The presence of *MiR-181* and *miR-17-5p* were observed in B- and T-cell lymphomas compared to non-neoplastic cells (128). Furthermore, miRNA dysregulation appears to have a key role in regulating metastasis in some canine cancer. The expression of 14 miRNAs were significantly different between metastasizing and non-metastasizing uveal melanomas, highlighting *cfa-miR-362*, *cfa-miR-155*, *cfa-miR-182*, and *cfa-miR-124* as strongly associated with the metastasizing class in this type of cancer (129). Ten miRNA (*cfa-let-7c*, *cfa-miR-10b*, *cfa-miR-26a*, *cfa-miR-26b*, *cfa-miR-29c*, *cfa-miR-30a*, *cfamiR-30b*, *cfa-miR-30c*, *cfa-miR-148a*, and *cfa-miR-299*) were validated and showed significant different expression in metastatic and non-metastatic mammary tumors (130). *MiR-9* was found to be overexpressed and associated with metastasis in mast cells tumors and osteosarcoma (121, 122), while *miR-34a* also appeared to be associated with invasion ability in canine osteosarcoma cell lines (131). Some miRNAs also correlated with tumor grading in canine splenic lymphoma (132).

Circulating miRNAs detected on liquid biopsies such as blood and urine may provide diagnostic and prognostic information regarding cancer (133). *MiRNA-214* and *-126* have been considered potential diagnostic and prognostic biomarkers for canine neoplastic diseases. In a recent study, using 181 cases of canine neoplastic diseases and healthy controls, circulating *miRNA-214* was considered a good diagnostic marker in sarcomas, whereas levels of the circulating *miRNA-126* was high in most of the types of canine tumors (134). These same miRNAs were demonstrated to have a strong potential to predict the outcome of canine appendicular osteosarcoma patients receiving amputation and chemotherapy (135). Dogs with disseminated histiocytic sarcoma and carcinomas showed downregulation of circulating *Let-7g* (136). Another study described the profile of circulating serum miRNAs in dogs with lymphoma. Four miRNAs (*let-7b*, *miR-223*, *miR-25*, and *miR-92a*) were significantly reduced in dogs with lymphoma, whereas *miR-423a* levels were significantly increased compared to the controls (137). *MiR-99a* was also differentially expressed in the plasma of dogs with lymphoma (125). Circulating miRNAs detected in the urine are also been detected in canine cancers. In canine bladder transitional cell carcinomas, *miR-103b* and *miR-16* were considered as potential diagnostic urine biomarkers (138). Analysis of the miRNA profiles within the exosomes released from canine tumors has also been made. A recent study observed that canine mammary epithelial cancer cells shed exosomes that contained differentially expressed miRNAs in comparison with normal cells (139). In a study performed in canine lymphoma, three miRNAs (*miR-151*, *miR-8908a-3p*, and *miR-486*) derived from exosomes demonstrated to be differently expressed between vincristine-sensitive and resistant lymphoma cell lines supporting a role of these miRNAs in the resistance of this cancer (140).

LncRNAs are non-coding transcripts greater than 200 bp in length and some studies demonstrated the influence of these molecules in gene expression at the epigenetic, transcriptional, and post-transcriptional levels. One of the most classical mechanisms through which lncRNAs regulate gene expression is by association with chromatin modeling complexes and transcription factors, influencing transcriptional repression and activation of gene promoters. For example, the well-characterized lncRNA *HOTAIR* can bind to epigenetic complexes such as PRC2 and LSD1/CoREST/REST, modulating histone methylation (141). In addition, lncRNAs binds directly to DNA, mRNAs, and/or miRNAs affecting and regulating their respective functions and levels (142–144).

The lncRNAs play a fundamental role in the development and physiology of the human organism but can be also associated with disease evolution, especially cancer. *HOTAIR* overexpression, for example, has been associated with an increase of metastasis, invasiveness, and, consequently, to poor outcomes in breast and other types of cancer (145, 146). Many oncogenic lncRNAs including *THOR* (147), *ARLNC1* (143), *SAMSOON* (148), and *EPIC1* have also been associated with different types of cancer such as lung, prostate, melanomas, ovarian, and pancreatic cancer (149).

Due to the importance of lncRNAs in the genome and their association with different human diseases, the canine lncRNA profile has also been described (150). An alignment-free program that accurately annotates lncRNAs, FEELnc, was used on a real data set of 20 RNA-Seq data from 16 different canine tissues produced by the European LUPA consortium to expand the canine genome annotation including 10.374 novel lncRNAs and 58.640 mRNAs transcripts (151). This study particularly highlighted duplications of lncRNAs in dog. Interestingly, among the novel lncRNAs genes, around 15% were also described as non-protein coding genes in the human GENCODE. Finally, with this set of data, it was possible to annotate three new cancer susceptibility candidate lncRNAs in dogs, which are well described in human cancer, including *CASC9*, associated with esophageal squamous cell carcinoma (152), *MALAT1*, associated with metastasis in lung cancer (153), and *IFNG-AS* that plays an important role in T-cell differentiation (154). Another study, observed more than 900 dog-human conserved lncRNAs using comparative genomics. The authors confirmed the annotation of well-studied lncRNAs in dogs, such as *HOTAIR*, *MALAT*, *NEAT_1*, *PCA3*, *CASC15*, *CASC17*, *CASC18*, *CASC20*, and *INHBA-AS1*. In addition, 44% of the canine lncRNAs are expressed in a tissue-specific manner, which is also widely seen in humans (155). Finally, co-expression analysis suggested that these lncRNAs function as regulatory elements in the dog genome (156). Despite the increase of the lncRNA number and description, few lncRNA are functionally and experimentally characterized in dogs, and only few of them have been found to be associated with diseases. For instance, the lncRNA *GDNF-AS* was observed to be involved in a Hereditary Sensory Autonomic Neuropathy (HSAN) in hunting dogs (157).

LncRNAs is also associated with some types of canine cancer. Cross-species analysis of lncRNAs demonstrated that a non-negligible fraction of lncRNA associated with human diffuse large B-cell lymphoma (DLBLC) is also expressed in canine lymphoma (155). A recent study has also developed a methodology to identify lncRNAs in canine DLBCL. The authors concluded that this methodology was able to quantify the expression of novel and annotated lncRNAs and, interestingly, subclassified the DLBCL in two main groups. Furthermore, these two DLBCL groups showed statistically different survival rates, pointing to the potential of using lncRNAs as prognostic markers using this methodology (158). In canine oral melanomas, 417 differentially expressed lncRNAs were identified in comparison with control samples, using deep transcriptome sequencing. Most of these lncRNAs have not yet been functionally characterized; however, lncRNA *ZEB2-AS*, a lncRNA involved in the regulation of the transcription factor *ZEB2* during epithelial-mesenchymal transition (EMT) in human colon, pancreatic, and breast cancer cell lines (159), was highly expressed in canine oral melanomas compared to control samples. Other examples of lncRNAs dysregulated in these tumors that are well described in human cancer, were *SOX21-AS1* (160), and *CASC15* (161). Finally, using co-expression network analysis (WGCNA), the differentially expressed lncRNAs were associated with Gene Ontology (GO) biological process including cancer-related genes, cell cycle, cellular response to stress, DNA metabolic process, and carbohydrate metabolism (162).

## Epigenetic Drugs to Treat Canine Cancer

Contrary to genetic mutations, epigenetic changes occurring in cancer are potentially reversible. There is thus the possibility of treating cancer with epigenetic drugs and, consequently, reverse some malignant phenotypes including metastasis potential (163, 164), tumorigenicity (165) and multidrug resistance (166). Several efforts have been undertaken for the development of epigenetic drugs targeting defective DNMTs and histone modifying enzymes as well as reader domains in cancer, but, unlike in human oncology, epigenetic drugs are still little in use in veterinary oncology, as we outlined below. However, it is important to emphasize that dogs are used as models in most preclinical tests for these drugs providing an overview of the possible side effects of these anticancer agents in this species.

## DNA Methyltransferases Inhibitors

The first two epigenetic cancer drugs, the 5-azacytidine (5-azaC or azacitidine) (167) and the 5-aza-2′-deoxycytidine (5-aza-dC or decitabine) (168) were synthesized in 1964 but only approved by the FDA in 2004 and 2006, respectively (169, 170). Both are DNMT inhibitors and are currently first-line therapy for myelodysplastic syndrome (MDS), a bone marrow disorder that can progress to acute myeloid leukemia (AML). Furthermore, 5-azaC and 5-aza-dC are administrated to treat hematological malignancies including chronic myelomonocytic leukemia (CMML) and AML in elderly patients ineligible for intensive chemotherapy (171–173). Although efficient, this first generation of DNMT inhibitors presents some issues including lack of specificity, which could trigger some side effects, poor bioavailability and limited half-life (174). Thus, second-generation DNMT inhibitors have been developed including zebularine (175), CP-4200 (176) and guadecitabine (177). For solid tumors, azanucleoside-based therapies are also being tested in phase I/II clinical trials in several types of cancer (178, 179).

Due to their promising results in human cancer, DNMT inhibitors have been tested, although at a low scale, in canine cancer (**Supplementary Table 4**). The first 5-aza-dC test report in dogs was published in 1983. Dogs were used to investigate the plasma and cerebrospinal fluid pharmacokinetics of 5-aza-dC and the results showed that the compound could be rapidly cleared from plasma and cross the blood-CSF barrier resulting in potentially and cytotoxic concentrations by infusion (180). However, in this study, dogs were only used as experimental models, not aiming treatment of canine cancer. Dogs with naturally occurring invasive urothelial carcinoma were treated with subcutaneous 5-aza-C. Of the 18 dogs in the study, partial remission was achieved in 22%; 50% showed stable disease, whereas in 22%, the cancer progressed. The subcutaneous 5-azaC strategy in dogs was considered promising and important for the translation and design of human urothelial carcinoma clinical trials (181).

In human and canine melanoma cells, a recent study has shown a new apoptosis-inducing mechanism of 5-aza-dC through demethylation and induction of cytotoxic cytokines such as TNF-α in *in vitro* and *in vivo* experiments, suggesting a potential therapeutic agent for human and canine melanomas (60). 5-aza-C reduced *in vitro* growth, invasion, tumorigenicity, and mitochondrial activity and increased the susceptibility to apoptosis of breast cancer cells from human, canine, and feline species. In addition, 5-aza-C was toxic to mammary cancer cells but not to healthy mammary cells lines from these species, indicating a therapeutic window and sustaining animals as useful models for pre-clinical evaluation of new drugs targeting breast cancer (182). Likewise, second-generation of DNMT inhibitors have been tested in canine models. Zebularine was able to inhibit DNMT1 and promote global demethylation of canine malignant lymphoid cells resulting in dose-dependent apoptosis (183). Toxicological and pharmacokinetic studies with Zebularine were performed using laboratory animals and dogs with natural occurring tumors. Plasma zebularine clearance was constant. Laboratory dogs treated with a daily oral zebularine dose of 4 mg kg^-1^ developed some side effects including neutropenia, found in all dogs, thrombocytopenia in one dog, anorexia in four dogs, and dermatological changes. In the dogs with tumors, thrombocytopenia was observed in one dog. No other hematologic abnormalities, serum biochemical abnormalities or dermatologic changes were detected. Despite important information regarding plasma pharmacokinetics and toxicity of zebularine in dogs, more studies should still be performed to observe the anticancer activity of zebularine in this specie (184).

## Histone Deacetylase Inhibitors (HDACi)

Since HDACs are often dysregulated in different types of cancer, many efforts have been made to develop efficient HDAC inhibitors. In human multiple myeloma, ovarian, gastric, breast, pancreatic, and prostate cancer, for example, HDACs are overexpressed and associated with poor outcome (185–192). Different patterns of HDAC1 have been established as prognostic marker in osteosarcoma. Whereas in primary osteosarcoma, cells showed a high expression of HDAC1 and 2, low levels of HDAC1 were associated with the presence of initial metastasis (193). Considering the relevance of this target class, four HDACs inhibitors have been approved for cancer treatment by the U.S Food and Drug Administration (FDA): vorinostat, romidepsin, belinostat, and panobinostat. The first HDACi, SAHA (Vorinostat, Zolinza™, Merck & Co, Inc., USA), was approved by FDA in 2006, and since then, HDACi are being developed for the treatment of T-cell lymphoma (194–196) and multiple myeloma (197). Furthermore, other studies and clinical trials showed the effects of Vorinostat in hematological and solid cancers including pancreatic (198), ovarian (199), prostate (200), and breast cancer (201).

HDACis have been also shown good effects in canine cancer (**Supplementary Table 5**). Vorinostat reduced the viability and increased apoptosis in a dose-dependent manner besides decreasing phosphorylation in oncogenic pathways including Akt-Ser^473^ and mTOR in canine osteosarcoma cell lines (202). In canine urothelial carcinoma cells, Vorinostat inhibited the growth and induced G0/G1 cell cycle arrest through the upregulation of p21 and dephosphorylation of Rb in these cancer cells (100). Both studies showed that Vorinostat was able to induce histone H3 acetylation in these canine cancer cells. The effects of another HDACi, sulforaphane, has been shown on canine osteosarcoma cells, significantly decreasing cell invasion and downregulating focal adhesion kinase (FAK) signaling (203).

A panel of seven HDACis were tested, in a well-established canine B-cell lymphoma cell line, CLBL-1 using *in vitro* and *in vivo* (xenograft) models. All HDACis tested exhibited dose-dependent inhibitory effects on the proliferation of CLBL-1 cells. Furthermore, Panobinostat, the most potent HDACi tested *in vitro*, inhibited CLBL-1 xenograft tumor growth, triggering acetylation of H3 and apoptosis *in vivo* (204). Panobinostat also efficiently inhibited the growth of tumors in xenograft models inoculated with a modified and bioluminescent canine B-cell lymphoma cell line (205). Trichostatin A (TSA), an antifugical agent with properties to selectively inhibit histone deacetylase activity in mammalian cells has shown inhibitory effects of proliferation and apoptosis in cancer cells (206). *In vitro* inhibitory effects of TSA were also shown on canine grade 3 mast cell tumor, decreasing cell viability, by increasing apoptosis and the number of cells in sub-G1 phase of cell cycle, indicating cell death (207). TSA also inhibited the proliferation of one canine mammary cancer cell line (208). A novel HDAC inhibitor AR-42, recently in phase I/Ib trials for multiple myeloma and T- and B-cell lymphomas (209), has shown effects in canine osteosarcoma, prostate, and malignant mast cancer cells. Cell viability inhibition and induction of apoptosis *via* activation of the intrinsic mitochondrial pathway were observed in canine osteosarcoma cells treated with AR-42. In addition, AR-42 showed synergistic effects when combined with doxorubicin (202). In canine prostate cancer, AR-42 inhibited *in vitro* proliferation in a time- and dose-dependent manner and decreased migration and the incidence of bone metastasis in xenograft models (210). AR-42 treatment of canine malignant mast cells induced proliferation inhibition, cell cycle arrest, apoptosis, and activation of caspases-3/7. Downregulation of KIT, a commonly mutated gene in malignant mast cells, *via* inhibition of KIT transcription was also observed. Finally, AR-42 treatment downregulated several important cancer molecules including p-AKT, total AKT, phosphorylated STAT3/5, and total STAT3/5 (211).

The effects of HDACs inhibition in combination with other therapies has also been studied in canine cancer. A phase I pharmacokinetic and pharmacodynamic study of combined valproic acid (VPA) and doxorubicin was performed in spontaneous canine cancers. Of the 21 dogs treated in this study, two presented complete responses (10%) (both lymphomas), three presented partial responses (14%) (lymphoma, melanoma, and lung carcinoma), five showed stable disease after treatment (24%) (osteosarcoma, renal cell carcinoma, apocrine gland adenocarcinoma, melanoma, and soft-tissue sarcoma), and 11 exhibited progressive disease (58%) (212). In another study from the same group using both human and canine osteosarcoma (OS) cells, pre-incubation with VPA followed by doxorubicin increased the growth inhibition and apoptosis rates in both human and canine OS cells, associated with a dose-dependent increase in nuclear doxorubicin accumulation, supporting a potential addition of HDACis for treatments of human and canine OS (213).

## Alternative Epigenetic Targets

Due to successful development of DNMT and HDAC inhibitors and the use of these molecules in the treatment of diseases, new classes of epigenetic drugs has been developed to target epigenetic writers, erasers and even epigenetic readers. Several examples are reported including histone methyltransferases inhibitors such as DOT1L (214), EZH2 (215), and G9A inhibitors (216). Many studies are also observing the effects of lysine demethylases inhibition including LSD1 and LSD2 (217) and epigenetic readers such as bromodomains with the BET (Bromo and Extra terminal) family comprising BRD2, BRD3, BRD4, and BRDT as the most prominent targets for drug discovery (218). During the past years, the Structural Genomic Consortium (SGC), a public-private partnership that supports the discovery of new medicines through open access research, has designed and developed a set of tool compounds for epigenetic targets with clearly defined properties (219, 220).

**Figure 3:**
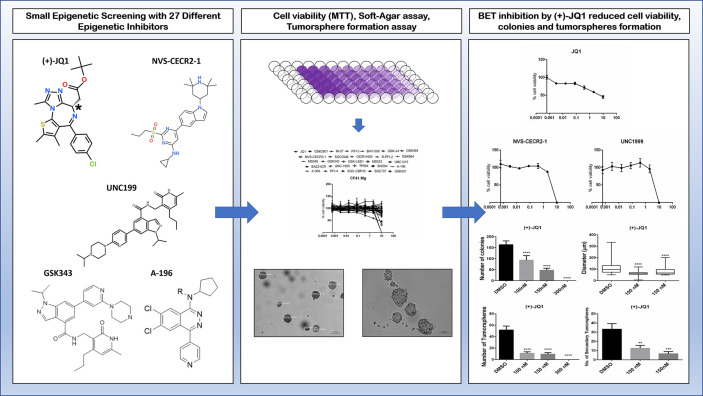
Effects of some alternative epigenetic inhibitors in canine mammary cancer cells. A small library of 27 epigenetic inhibitors was screened in order to determine effects regarding cell viability, tumorigenicity, and self-renewal assessed by 3D cell culture models such as colony formation in soft-agar and tumorspheres formation in low-adherent plates (221). The (+)-JQ1 (BET family inhibitor), NVS-CECR2-1 (CECR2 inhibitor), and UNC1999 (EZH2/1 inhibitor) decreased cell viability of CF41.Mg canine mammary cancer cell line. Furthermore, (+)-JQ1 exhibits a strong impact on colony and tumorspheres formation, demonstrating effects on tumorigenicity and self-renewal phenotypes (222).

Our group recently screened a small-molecule library containing 27 of these developed epigenetic inhibitors in canine mammary cancer cell lines (CMCs). We observed three inhibitors inducing significant reduction of cell viability in CMCs including (+)-JQ1 (BET family inhibitor), NVS-CECR2-1 (CECR2 inhibitor), and UNC1999 (EZH2/1 inhibitor). Furthermore, BET inhibition by (+)-JQ1 was very efficient to inhibit CMCs colony and tumorsphere formation, demonstrating an effect on tumorigenicity and self-renewal (**Figure 3**) (222). Inhibition of SET methyltransferase by shRNA and FTY720, reported to directly interact with SET proteins, suppressed cell proliferation, colony formation, and *in vivo* tumor growth of canine mammary and osteosarcoma cell lines. Furthermore, SET knockdown repressed mTOR and NF-kB signaling in both types of canine cancer (223, 224). Using BB-Cl-Amidine (BB-CLA) to inhibit protein-arginine deiminases (PADs) resulted in the decrease of viability and tumorigenicity of canine mammary cancer cells, activating endoplasmic reticulum stress pathway in these cells (225).

Dogs have also been used as models for pre-clinical trials of LSD1 inhibitors. A recent study, showed that the LSD1 inhibitor GSK2879552 caused severe but reversible toxicities in dogs including thrombocytopenia, neutropenia, myelofibrosis, and congestion with and without lymphoid necrosis in lymphoid organs (217). However, studies demonstrating the effects of these new alternative epigenetic drugs in dogs are very scarce (**Supplementary Table 6**).

## BarkBase: A Canine Epigenomic Resource

The abundance of information acquired in recent years on human genomics both in healthy and diseased tissues enabled the construction of powerful platforms of data that can be used for the deep investigation of different phenotypes. Resources such as ENCODE (226), GTEx (227), Cancer Genome Atlas (TCGA) (https://www.cancer.gov/tcga) and NIH reference human epigenome (228), are constantly fueled with large numbers of information generated with next-generation bioinformatic tools and are extremely important for the elucidation of the most diverse diseases that affect humans and their therapeutic advances. With the same purpose, a ~7 gigabytes genomic data platform, the BarkBase resource, has been recently developed (229). BarkBase contains data for 27 adult tissue types, with biological replicates, from five adult dogs, paired with 30x whole genome sequence data. RNA sequencing data are complemented by whole genomic sequencing and assay data for transposable-accessible chromatin using sequencing (ATAC-seq). All these genomic and epigenomic data from healthy canine tissues will be highly important and useful for future studies in canine cancer providing the basis of a high-quality tool to compare the findings found in canine cancer tissues with healthy tissues. Furthermore, BarkBase introduces a reliable and solid resource to support comparative studies between canine and human species (http://www.barkbase.org/).

## Conclusion and Perspectives

Epigenetic alterations are present and possibly regulating several types of canine cancer. Furthermore, many of these epigenetic alterations in canine cancer are also observed in human cancer including genome-wide hypomethylation, hypermethylation of tumor suppressor genes, aberrant histone modifications, and dysregulation of non-coding RNA (**Table 1**). These data suggest a potential approach using the canine model to determine new epigenetic mechanisms regulating cancer, diagnostic/prognostic markers, and targets for the development of new anticancer drugs. Interestingly, these findings increase the possibility to investigate which environmental factors play a role in epigenetic alterations both in human and dogs, since both species are exposed to the same carcinogens in the environments during their life, and, surprisingly, few studies aimed to observe the environmental risk factors in canine cancer (236–240). Despite these similarities, some differences regarding the epigenetic landscape can be observed in human and canine cancer such as the epigenetic regulation of estrogen receptor α between breast cancer in women and mammary cancer in dogs and the epigenetic regulation of ABCB1 gene in lymphomas. In addition, most studies aiming to elucidate the epigenetic profile of canine cancer and to determine possible targets and therapies for this disease in these animals are performed using *in vitro* models. Thus, further investigations are needed to confirm the potential of use dogs as a comparative and translational model to study epigenetics.

**Table 1:** Comparative studies regarding epigenetics changes in different human and canine cancer.

Human/Dog comparative epigenetic studies			
Associated epigenetic modification	Findings	Type of tumor	Reference
DNA Methylation	Genomic hypomethylation has been observed in Human and canine lymphoma and leukemia	Lymphoma; Leukemia	(49) x (230)
DNA Methylation	Hypermethylation of Tumor suppressor gene *DLC1*	Non-hodgkin’s Lymphoma	(57) x (58)
DNA Methylation	Treatment with 5-AzaC reduces tumorigenicity in mammary cancer cells of Human, Dogs and cats.	Mammary Cancer	(182)
DNA Methylation	Methylation levels of *LINE-1* in circulating cell-free DNA (cfDNA) might be a useful diagnostic marker in human and canine mammary cancer.	Mammary Cancer	(231)
DNA Methylation	DNA methylation of microRNA-203 CpG islands contributes to Human and Canine Melanoma	Melanoma	(61)
DNA Methylation	Hypermethylation of *TNF-α* promoter region was identified in human and canine melanoma cancer cells. Furthermore, the study observed a novel apoptosis-inducing mechanism of 5-aza-2-deoxycitidine.	Melanoma	(60)
DNA Demethylation	*TET2* is commonly mutated in human hematopoietic tumors. However, the *TET2* mutation frequency in canine hematopoietic tumors, such as mast cell tumor and lymphomas, is very low.	Hematopoietic tumors	(80) x (81) x (83)
DNA Methylation and histone modification	Combination of DNA methylation inhibitors and Chromatin-modified drugs is promising in Human and canine Osteosarcoma.	Osteosarcoma	(232)
Histone modifications	The HDAC inhibitor valproic acid can be used in combination with doxorubicin to treat human and canine osteosarcoma	Osteosarcoma	(213)
Histone modifications	HDAC inhibitor AR-42 induce apoptosis both in human and canine osteosarcoma cells.	Osteosarcoma	(202)
MicroRNAs	The role of miRNAs in human and mammary cancer.	Mammary cancer	(233)
MicroRNAs	MicroRNAs as tumor suppressors in human and canine melanoma cells	Melanoma	(234)
MicroRNAs	Antioncogenic miRNA-145 was downregulated in both human and canine melanoma cells	Melanoma	(235)
LncRNAs	Oncogenic lncRNAs in human cancer, including *HOTAIR*, *MALAT1*, *PCA3*, *CASC15*, and *CASC20* are also annotated in dogs.	Different types of tissues	(151) x (156) x (150)
LncRNAs	A cross-species analysis of lncRNAs demonstrated that lncRNA associated with human diffuse large B-cell lymphoma (DLBLC) is also expressed in canine lymphoma	Lymphoma	(155)
LncRNAs	LncRNA *ZEB2-AS*, *SOX21-AS1*, and *CASC15*, well-described in human cancer, was highly dysregulated in canine oral melanomas.	Melanoma	(159) x (160) x (161) x (162)

Currently, therapeutic options to treat canine cancer are basically surgery, radiotherapy, hyperthermia, photodynamic therapy, immunotherapy, and chemotherapy (241). Thus far, there is no epigenetic drug specific to treat canine cancer or being used in veterinary oncology clinics. This fact is probably a consequence of the lack of solid studies determining the main epigenetic targets in canine cancer. Studies of targeted therapy in dogs using appropriate protocols and models inhibiting epigenetic targets are missing to investigate the potential of epigenetic modulation for the treatment of canine cancer in clinics. Furthermore, all current epigenetic drugs were designed for human treatment. Thus, despite promising *in vitro* results of epigenetic drugs in canine cancer cells, the effect of the compounds was not optimized for canine cancers, and the side effects may be present due to differences in physiology between man and dog.

Following the exciting development of studying the role of epigenetic reprogramming in human cancer, this area is also emerging in veterinary oncology. Several studies have unveiled epigenetic alterations in canine cancer types, and importantly, some common features corroborate findings observed in human cancer. There are several important similarities such as spontaneous tumor development and the influence of environmental factors that entail for more thoroughly designed comparative studies of human and dog cancer. Databases such as CCOGC (Canine and Comparative Oncology and Genomics Consortium) and BarkBase provide promising first steps and tools to elucidate the mechanisms behind canine cancer and support comparative studies between dogs and humans. However, these are only first steps and more research is necessary in order to better understand dogs as models to study epigenetics in cancer and drug development. We hope that the advancement of knowledge and technologies of epigenetic tools will aid the development of new targets and the advancement of drugs in the area of veterinary oncology.

## Author Contributions

PX performed the literature review, wrote the manuscript, and produced figures and tables. SM wrote and reviewed the manuscript, figures, and tables. HF reviewed the manuscript. All authors contributed to the article and approved the submitted version.

## Funding

This review has been supported by grants from the Sao Paulo Research Foundation (FAPESP) (grant and PX scholarship numbers: 2014/02493-7, 2017/11966-4, and 2019/05778-6). SM is grateful for funding received from the SGC, a registered charity (no: 1097737) that receives funds from; AbbVie, Bayer AG, Boehringer Ingelheim, Canada Foundation for Innovation, Eshelman Institute for Innovation, Genentech, Genome Canada through Ontario Genomics Institute [OGI-196], EU/EFPIA/OICR/McGill/KTH/Diamond, Innovative Medicines Initiative 2 Joint Undertaking [EUbOPEN grant 875510], Janssen, Merck KGaA (aka EMD in Canada and US), Merck & Co (aka MSD outside Canada and US), Pfizer, São Paulo Research Foundation-FAPESP, Takeda and Wellcome [106169/ZZ14/Z].

## Conflict of Interest

The authors declare that the research was conducted in the absence of any commercial or financial relationships that could be construed as a potential conflict of interest.
