# Supplementary material for: Epigenetic Mechanisms in Canine Cancer
Source: Front Oncol. 2020 Oct 23;10:591843. doi: 10.3389/fonc.2020.591843 (PMC7646326; doi:10.3389/fonc.2020.591843)
Supplement: Supplementary file 1 [file Table_1.docx]

**Supplementary Tables**

**Supplementary Table 1.** DNA Methylation modifications associated with different types of canine cancer.

| **DNA Methylation and Canine Cancer** | | | |
| --- | --- | --- | --- |
| **Associated Epigenetic Modification** | **Findings** | **Type of Tumor** | **Reference** |
| DNA Hypomethylation | Genomic hypomethylation has been observed in canine lymphoma and leukemia | Lymphoma; Leukemia | [50] |
| DNA Hypomethylation | Genome hypomethylation was found in grade III canine mast cell tumor, correlating with the aggressiveness of this type of cancer | Mast cell tumor | [54] |
| DNA Hypomethylation | Higher DNA global hypomethylation of circulating leukocytes in dogs with NHL in comparison with healthy dogs | Non-Hodgkin Lymphoma | [55] |
| DNA Hypomethylation | DNA hypomethylation observed in canine lung cancer and in metastatic osteosarcoma from the primary lung cancer | Lung cancer | [56] |
| DNA Hypermethylation | DNA hypermethylation in DLC1, a tumor suppressor gene | Non-Hodgkin Lymphoma and canine chronic lymphocytic leukemia | [59] |
| DNA Hypermethylation | DLC1 hypermethylation was associated with the malignant phenotype of NHL. However, hypermethylation of the DLC1 promoter was not associated with silencing of DLC1 expression and did not correlate with survival | Non-Hodgkin Lymphoma | [59;60] |
| DNA Hypermethylation | Hypermethylation of *TNF-α* in canine melanoma cells. The methylation status and the level of *TNF-α* expression were inversely correlated in canine melanoma cell lines and melanoma tissues | Melanoma | [61] |
| DNA Hypermethylation | DNA hypermethylation of microRNA-203 CpG islands, a tumor suppressor miRNA | Melanoma | [62] |
| DNA Hypermethylation | DNA Hypermethylation of *TFPI-2* | B-cell Lymphoma | [64] |
| DNA Hypermethylation | DNA hypermethylation of DAPK | B-cell Lymphoma | [65] |
| DNA Hypermethylation | DNA hypermethylation of p16 | Lymphoid Tumor | [66] |
| DNA Hypermethylation | DNA hypermethylation of HOXD10, FGFR2, ITIH5, RASAL3 | B-cell Lymphoma | [67;68] |
| DNA Hypermethylation | *DAPK* hypermethylation is a negative prognostic factor in canine high-grade B-cell Lymphoma. | B-cell Lymphoma | [69] |
| DNA Hyper/Hypomethylation | A heterogeneous pattern of DNA methylation observed with subsets of cases hypermethylated or hypomethylated when compared with healthy tissues. | Acute myeloid leukemia | [70] |
| DNA Hyper/Hypomethylation | CpGs hypermethylation located in CGIs that were unmethylated in normal peripheral blood mononuclear cells (PBMCs). CpG sites outside CGIs lose methylation in lymphoma cells compared to the healthy PBMCs. | Lymphoma | [71] |
| DNA Hypermethylation | *BRCA1* hypermethylation in mammary cancer. The rate of *BRCA1*-hypermethylated samples was very low (1/15 – 6.7%) making it difficult to conclude that *BRCA1* downregulation is a consequence of *BRCA1* promoter hypermethylation | Mammary cancer | [74] |
| DNA Hypermethylation | CGIs of the *ABCB1* gene were hypomethylated in dogs with lymphoma | Lymphoma | [75;77] |
| DNA Methylation | No variation in methylation patterns were found between *ERα*-positive canine mammary carcinomas and *ERα*-negative canine mammary carcinomas pointing to a difference of *ERα* regulation mechanisms between human and dogs. | Mammary cancer | [80] |
| TET-mediated DNA demethylation | In hematopoietic canine tumors, *TET2* mutations have also been observed in canine mast cell tumors but in very low frequency. | Mast Cell tumor | [82] |
| TET-mediated DNA demethylation | *TET2* is mutated in Canine T-cell lymphoma samples, but in low frequency. | Lymphoma | [84] |

**Supplementary Table 2.** Histone modifications associated with different types of canine cancer.

| **Histone Modifications and Canine Cancer** | | | |
| --- | --- | --- | --- |
| **Associated Epigenetic Modification** | **Findings** | **Type of Tumor** | **Reference** |
| Histone Deacetylation | Lower histone acetylation levels were associated with poor prognosis of the animals with urothelial carcinomas | Urothelial Carcinomas | [101] |
| Histone Methyltransferase Mutation | *SETD2* gene, a histone methyltransferase and an important tumor-suppressor, was found to be mutated in 21% of canine osteosarcoma samples. | Osteosarcoma | [102] |
| Histone Methyltransferase Mutation | *SETD2* somatic point mutations, deletions and chromosomal translocations in 42% of canine osteosarcoma samples. | Osteosarcoma | [103] |
| Histone Methyltransferase Overexpression | Overexpression of *EZH2* in different types of canine cancer | Lymphoma; melanoma; basal cell tumors; squamous cells carcinoma; prostate; and mammary | [104;105] |
| Histone Modification | Complex canine mammary carcinomas displayed a number of epigenetic dysregulations, such as downregulation of chromatin-modification genes or abnormally enriched activating histone modification H4-acetylation, and reduction in the repressive histone modification H3K9me3. | Mammary Cancer | [107] |

**Supplementary Table 3.** Non-Coding RNA abnormalities associated with different types of canine cancer.

| **Non-Coding RNA Modifications and Canine Cancer** | | | |
| --- | --- | --- | --- |
| **Associated Epigenetic Modification** | **Findings** | **Type of Tumor** | **Reference** |
| Abnormal miRNA expression | *miR-29* and *miR-29b* were upregulated in canine mammary cancer. *miR-15a* and *miR-16* are downregulated in canine ductal carcinomas while *miR-181b, -21, -29b*, and *let-7f* showed a significant upregulation in canine tubular papillary carcinomas | Mammary Cancer | [121] |
| Abnormal miRNA expression | *miR-9* is increased in high grade canine mast cell tumor compared to low grade and normal samples | Mast cell tumor | [122] |
| Abnormal miRNA expression | *miR-9* overexpression in osteosarcoma tumors and cell lines compared to normal osteoblasts and associated with metastatic phenotype | Osteosarcoma | [123] |
| Abnormal miRNA expression | 22 miRNAs are differentially expressed in splenic hemangiosarcoma and normal spleens samples | Hemangiosarcoma | [124] |
| Abnormal miRNA expression | 5 miRNAs upregulated and 14 downregulated in prostate cancer in comparison with normal prostate suggesting these miRs have diagnostic and miR-based therapeutic potential in prostate cancer | Prostate Cancer | [125] |
| Abnormal miRNA expression | Upregulation of the miR-17-92, miR-29 family, and miR-34a were associated with B cell lymphoma and upregulation of the miR-181 with T cell lymphoma. Upregulation of the miR-181 and downregulation of miR-29b and miR-150 were associated with inferior response to CHOP therapy and survival. Higher expression of miR-155 and miR-222 were negatively associated with outcome in both B and T cell lymphoma | Lymphoma | [126] |
| Abnormal miRNA expression | miR‑383 and miR‑204 were potential oncomiRs that may be involved in regulating melanoma development by evading DNA repair and apoptosis | Melanoma | [127;128] |
| Abnormal miRNA expression | Upregulation of m*iR-181* and *miR-17-5p* in B- and T-cell lymphomas | Lymphoma | [129] |
| Abnormal miRNA expression | *cfa-miR-362, cfa-miR-155, cfa-miR-182*, and *cfa-miR-124* as strongly associated with the metastasizing class in uveal melanomas | Melanoma | [130] |
| Abnormal miRNA expression | Ten miRNA were validated and showed significant different expression in metastatic and non-metastatic mammary tumors | Mammary cancer | [131] |
| Abnormal miRNA expression | *miR-34a* associated with invasion ability in canine osteosarcoma cell lines | Osteosarcoma | [132] |
| Abnormal miRNA expression | Circulating *miRNA-214* is a good diagnostic marker in sarcomas, whereas circulating *miRNA-126* was high in different types of canine tumors | Different epitelial and non-epithelial canine cancers. | [134] |
| Abnormal miRNA expression | miR-214 and miR-216 have a strong potential to predict the outcome of canine appendicular osteosarcoma patients receiving amputation and chemotherapy | Osteosarcoma | [136] |
| Abnormal miRNA expression | Downregulation of circulating *Let-7g* in histiocytic sarcoma | Histiocytic Sarcoma | [137] |
| Abnormal miRNA expression | Studying the profile of circulating serum miRNAs in dogs with lymphoma, *let-7b, miR-223, miR-25, miR-92a* were reduced, whereas *miR-423a* levels were significantly increased in dogs with lymphoma compared to the controls | Lymphoma | [138] |
| Abnormal miRNA expression | *miR-103b* and *miR-16* as potential diagnostic urine biomarkers to bladder cancer | Bladder cancer | [139] |
| Abnormal miRNA expression | Mammary cancer cells shed exosomes that contained differentially expressed miRNAs in comparison with normal cells | Mammary Cancer | [140] |
| Abnormal miRNA expression | *miR-151, miR-8908a-3p, and miR-486* derived from exosomes demonstrated to be differently expressed between vincristine-sensitive and resistant lymphoma cell lines. | Lymphoma | [141] |
| Abnormal lncRNA expression | Description of the canine lncRNA profile | Healthy Tissues | [151] |
| Abnormal lncRNA expression | Annotation of three new cancer susceptibility candidate lncRNAs in dogs, which are well described in human cancer, including *CASC9*, *MALAT1*, and *IFNG-AS.* | Sixteen different types of tissues | [152] |
| Abnormal lncRNA expression | LncRNAs associated with human diffuse large B-cell lymphoma (DLBLC) are also expressed in canine lymphoma. | Lymphoma | [156] |
| Abnormal lncRNA expression | More than 900 lncRNAs are conserved in humans and dogs. This study annotated well-studied lncRNAs in dogs, such as *HOTAIR*, *MALAT*, *NEAT_1*, *PCA3*, *CASC15*, *CASC17*, *CASC18*, *CASC20*, and *INHBA-AS1*. In addition, 44% of the canine lncRNAs are expressed in a tissue-specific manner, which is also widely seen in humans. | Twenty-six different types of tissues | [157] |
| Abnormal lncRNA expression | A new methodology to identify lncRNAs in canine DLBCL. The authors concluded that this methodology was able to quantify the expression of novel and annotated lncRNAs and, interestingly, subclassified the DLBCL in two main groups. Furthermore, these two DLBCL groups showed statistically different survival rates, pointing to the potential of using lncRNAs as prognostic markers. | Lymphoma | [159] |
| Abnormal lncRNA expression | In canine oral melanomas, 417 differentially expressed lncRNAs were identified in comparison with control samples. Some of these are well-studied in human cancer including *ZEB2-AS*, *SOX21-AS*, and *CASC15.* | Melanoma | [163] |

**Supplementary Table 4.** Studies observing the effects of DNA methyltransferases inhibitors in different types of canine cancer.

| **DNA Methyltransferase inhibitors and Canine Cancer** | | | |
| --- | --- | --- | --- |
| **Inhibitor** | **Findings** | **Type of Tumor** | **Reference** |
| 5-Aza-C | Dogs with urothelial carcinoma were treated with subcutaneous 5-aza-C. Partial remission was achieved in 22%; 50% showed stable disease, whereas in 22% the cancer progressed. | Urothelial Carcinoma | **[**182**]** |
| 5-Aza-dC | 5-aza-dC induced a new apoptosis-inducing mechanism in melanoma cells, through demethylation and induction of cytotoxic cytokines such as TNF-α in *in vitro* and *in vivo* experiments. | Melanoma | **[**61**]** |
| 5-Aza-C | 5-aza-C reduced *in vitro* growth, invasion, tumorigenicity, mitochondrial activity and increased the susceptibility to apoptosis of mammary cancer cells from human, canine and feline species. | Mammary cancer | **[**183**]** |
| Zebularine | Zebularine promotes global demethylation of canine malignant lymphoid cells resulting in dose-dependent apoptosis. | Lymphoma | **[**184**]** |

**Supplementary Table 5.** Studies observing the effects of histone deacetylase inhibitors in different types of canine cancer.

| **Histone Deacetylase Inhibitors and Canine Cancer** | | | |
| --- | --- | --- | --- |
| **Inhibitor** | **Findings** | **Type of Tumor** | **Reference** |
| Vorinostat | Vorinostat reduced the viability and increased apoptosis in a dose-dependent manner besides decreasing phosphorylation in oncogenic pathways including Akt-Ser^473^ and mTOR in canine osteosarcoma cell lines | Osteosarcoma | [203] |
| Vorinostat | Inhibited the growth and induced G0/G1 cell cycle arrest through the upregulation of p21 and dephosphorylation of Rb in urothelial carcinoma cells | Urothelial Carcinoma | [101] |
| Sulforaphane | Decreased cell invasion and downregulating focal adhesion kinase (FAK) signaling in canine osteosarcoma cells | Osteosarcoma | [204] |
| Panobinostat | Inhibited B-cell Lymphoma xenograft tumor growth, triggering acetylation of H3 and apoptosis in *vivo* | Lymphoma | [205] |
| Panobinostat | Panobinostat efficiently inhibited the growth of tumors in xenograft models inoculated with a canine B-cell lymphoma cell line | Lymphoma | [206] |
| Trichostatin A | Trichostatin A decreased cell viability and increased apoptosis in canine grade 3 mast cell tumor | Mast cell tumor | [208] |
| Trichostatin A | Trichostatin inhibited the proliferation of one canine mammary cancer cell line | Mammary Cancer | [209] |
| AR-42 | Induced cell viability inhibition and induction of apoptosis via activation of the intrinsic mitochondrial pathway were observed in canine osteosarcoma cells | Osteosarcoma | [203] |
| AR-42 | Inhibited *in vitro* proliferation in a time- and dose-dependent manner and decreased migration and the incidence of bone metastasis in xenograft models | Prostate Cancer | [211] |
| AR-42 | AR-42 in canine malignant mast cells induced proliferation inhibition, cell cycle arrest, apoptosis, and activation of caspases-3/7. Inhibition of KIT, p-AKT and STAT3/5 was also observed | Mast cells tumor | [212] |
| Valproic Acid (VPA) | A study of combined valproic acid (VPA) and doxorubicin was performed in spontaneous canine cancers. 10% presented complete responses, 14% presented partial responses, 24% showed stable disease after treatment, and 58% exhibited progressive disease | Lymphomas, melanoma, lung cancer, osteosarcoma, renal cell carcinoma, soft-tissue carcinoma. | [213] |
| Valproic Acid (VPA) | Pre-incubation with VPA followed by doxorubicin increased the growth inhibition and apoptosis rates in canine osteosarcoma cells | Osteosarcoma | [214] |

**Supplementary Table 6.** Studies observing the effects of alternative epigenetic inhibitors in different types of canine cancer.

| **Alternative epigenetic inhibitors and Canine Cancer** | | | |
| --- | --- | --- | --- |
| **Inhibitor** | **Findings** | **Type of Tumor** | **Reference** |
| (+)-JQ1 | (+)-JQ1, a BET proteins inhibitor, decreased cell viability of canine mammary cancer cells. Furthermore, (+)-JQ1 was very efficient to inhibit colonies and tumorspheres formation, demonstrating an effect on tumorigenicity and self-renewal | Mammary cancer | **[**222**]** |
| FTY720 | FTY720, a SET methyltransferase inhibitor, suppressed cell proliferation, colony formation, and *in vivo* tumor growth of canine mammary and osteosarcoma cell lines. Furthermore, SET knockdown repressed mTOR and NF-kB signaling in both types of canine cancer | Osteosarcoma; Mammary cancer | **[**223;224**]** |
| BB-Cl-Amidine | BB-CLA, an inhibitor of protein-arginine deiminases (PADs), resulted in the decrease of viability and tumorigenicity of canine mammary cancer cells, activating endoplasmic reticulum stress pathway in these cells | Mammary cancer | **[**225**]** |
| GSK2879552 | GSK2879552, a LSD1 inhibitor, caused severe but reversible toxicities in dogs including thrombocytopenia, neutropenia, myelofibrosis, and congestion with and without lymphoid necrosis in lymphoid organs | Healthy dogs used as preclinical models | **[**218**]** |
